# A Guideline for Open-Source Tools to Make Medical Imaging Data Ready for Artificial Intelligence Applications: A Society of Imaging Informatics in Medicine (SIIM) Survey

**DOI:** 10.1007/s10278-024-01083-0

**Published:** 2024-04-01

**Authors:** Sanaz Vahdati, Bardia Khosravi, Elham Mahmoudi, Kuan Zhang, Pouria Rouzrokh, Shahriar Faghani, Mana Moassefi, Aylin Tahmasebi, Katherine P. Andriole, Peter Chang, Keyvan Farahani, Mona G. Flores, Les Folio, Sina Houshmand, Maryellen L. Giger, Judy W. Gichoya, Bradley J. Erickson

**Affiliations:** 1 Artificial Intelligence Laboratory, Department of Radiology, Mayo Clinic, 200 1st Street, SW, Rochester, MN 55905 USA; 2 Department of Radiology, Thomas Jefferson University, Philadelphia, PA USA; 3 Department of Radiology, Brigham and Women’s Hospital, Harvard Medical School, Boston, MA USA; 4 Department of Radiological Sciences, Irvine Medical Center, University of California, Orange, CA USA; 5 Center for Biomedical Informatics and Information Technology, National Cancer Institute, Bethesda, MD USA; 6 NVIDIA, Santa Clara, CA USA; 7 Diagnostic Imaging & Interventional Radiology Moffitt Cancer Center, Tampa, FL USA; 8 Department of Radiology and Biomedical Imaging, University of California San Francisco, San Francisco, CA USA; 9 Department of Radiology, The University of Chicago, Chicago, IL USA; 10 Department of Radiology, Emory University School of Medicine, Atlanta, GA USA

**Keywords:** Artificial intelligence, Open source, Data curation, Toolkits

## Abstract

In recent years, the role of Artificial Intelligence (AI) in medical imaging has become increasingly prominent, with the majority of AI applications approved by the FDA being in imaging and radiology in 2023. The surge in AI model development to tackle clinical challenges underscores the necessity for preparing high-quality medical imaging data. Proper data preparation is crucial as it fosters the creation of standardized and reproducible AI models while minimizing biases. Data curation transforms raw data into a valuable, organized, and dependable resource and is a fundamental process to the success of machine learning and analytical projects. Considering the plethora of available tools for data curation in different stages, it is crucial to stay informed about the most relevant tools within specific research areas. In the current work, we propose a descriptive outline for different steps of data curation while we furnish compilations of tools collected from a survey applied among members of the Society of Imaging Informatics (SIIM) for each of these stages. This collection has the potential to enhance the decision-making process for researchers as they select the most appropriate tool for their specific tasks.

## Introduction

Artificial intelligence (AI) continues to play a significant role in medical imaging. As of 2023, the highest percentage of AI algorithms cleared by the FDA were for imaging (83%) and radiology (75%) [1, 2]. The increasing rate of AI model development to address clinical challenges has escalated the need to prepare high-quality medical imaging data. Optimal data preparation is of paramount importance since it leads to the development of standard, reproducible AI models and alleviates biases [3].

Data curation for AI model development is a multifaceted and challenging process. Data curation creates a dataset representative of the problem domain. Crucially, the representativeness of the data directly influences the performance and generalization capabilities of the AI models [4]. Effective data curation ensures that raw data is transformed into a high-quality, organized, and reliable resource that underpins the success of machine learning and analytical endeavors [5]. The ideal tool for data curation should assist developers and researchers in preparing the data in the fastest and most well-curated manner. Such tools not only save time but also contribute to the accuracy and robustness of models. Staying well-versed in these tools empowers professionals to navigate the complex journey from raw data to refined information, thus unlocking the true potential of data-driven innovations [6]. Given the abundance of tools, each targeting distinct aspects of data preparation but often sharing considerable similarities, it is crucial for researchers and developers to remain well-informed about the most applicable tools within their particular research domains.

Gaining knowledge regarding available tools not only helps selecting the best tool for the assigned task (e.g., detection, segmentation, or classification) but also can point out the possible limitations the user might face if starting to work with the inappropriate tool (for instance a tool that can only create one label in multi-label segmentation task).

In the current study, we aim to provide a descriptive outline for the phases of data curation while we furnish compilations of tools gathered from a survey carried out among members of the Society of Imaging Informatics (SIIM) for each of these stages. This compilation serves to enhance the decision-making process for researchers as they select the suitable tool for their specific tasks.

## Method

For data collection, a survey consisting of questions requesting researchers to identify their preferred tool, provide a description of the tool, and highlight its core features was created and shared with 500 members of the Society of Imaging Informatics (SIIM). A total of 54 responses from 26 medical informatics centers were collected. Duplications, general answers that did not introduce a specific tool, not open-access, and in-house solutions (not publicly available) were excluded, resulting in the inclusion of a total of 28 tools. The tools in the next phase were carefully investigated by the authors (S.V, B.K, E.M, P.R, S.F, M.M, A.T) to be characterized based on the core features, including cloud features, input data, de-identification functions, data conversion, data normalization, data labeling, data annotation, storage, workflow, and federated learning support. In the subsequent sections, we briefly describe different steps of data curation with a collected list of tools that are particularly useful for each task (Tables 1 and 2). References and links to all tools are given in the supplemental material. We created the SIIM Tools Survey GPT (https://chat.openai.com/g/g-X6o0w5duF-siim-tools-survey) using GPT4 as a chatbot based on the collected information.

**Table 1:** Tools used for data curation with their specific core features

**Tool**	**De-identification**	**Viewer**	**Cloud-based**	**Input/output**	**Conversion**	**Normalization**
3D slicer	✓	✓	Both^1^	DCM^2^, NIfTI/DCM, NIfTI	✓	✓
Anonymizer (RSNA)	✓			DCM/DCM + spreadsheet		
CTP (Clinical trial processor)	✓	✓		DCM/DCM		
DICOM image analysis and archive (DIANA)	✓		Both	DCM/DCM		
dicom2nifti				DCM/NIfTI	✓	
DCM2niix	✓			DCM/NIfTI	✓	
Highdicom			Both	Numpy/DCM	✓	
Horos	✓	✓	Both	DCM/DCM		
ImageJ/FIJI	✓	✓		DCM, NIfTI/DCM, NIfTI	✓	✓
ITK-SNAP		✓		DCM, NIfTI, PNG, JPEG/NIfTI	✓	✓
MANGO		✓		DCM, NIfTI/NIfTI	✓	✓
MIDRC	✓	✓	✓	DCM, DCM mapping/attribute		✓ (Harmonization)
The Medical Imaging Interaction Toolkit (MITK)		✓		DCM, NIfTI/DCM, NIfTI	✓	✓
MONAI label			Both	DCM, NIfTI/DCM, NIfTI	✓	✓
MOOSE				DCM/NIfTI	✓	✓
Niffler	✓			DCM/NIfTI	✓	

“Both” indicate cloud-based and non-cloud-based features. “*DCM*” is an abbreviation of DICOM files

**Table 2:** Tools used for data annotation with their specific core features

**Tool**	**Cloud base**	**Input/output**	**Labeling**	**Segmentation**	**Active learning**	**Object detection**	**3D rendering**	**Co-registration**	**Classification**
3D slicer	Both^1^	DCM^2^, NIfTI/DCM, NIfTI	✓	✓ + auto segmentation	✓		✓	✓	
Computer vision annotation tool	Both	JPEG, PNG/Json		✓	✓	✓			
Horos	Both	DCM/database in text files with comments or notes	✓						
ImageJ		TIFF, PNG, JPEG, DCM, FITS	✓	✓			✓	✓	
ImageJ/FIJI		DCM, NIfTI/DCM, NIfTI	✓	✓		✓	✓	✓	✓
ITK-SNAP		DCM, NIfTI, PNG, JPEG/NIfTI	✓	✓ + auto segmentation		✓	✓	✓	
Labelme	Both	JPEG/JSON	✓	✓	✓	✓			
MANGO		DCM, NIfTI/NIfTI	✓				✓		
Markit		DCM/CSV	✓			✓			✓
MONAI label	Both	DCM, NIfTI/DCM, NIfTI	✓	✓ + auto segmentation	✓				✓
The Medical Imaging Interaction Toolkit (MITK)		DCM, NIfTI/DCM, NIfTI		✓ + auto segmentation			✓	✓	
MOOSE		DCM/NIfTI (3D)	✓	✓ + auto segmentation					
NCI Imaging Data Commons (IDC)	✓	DCM, NIfTI/DCM, NIfTI	✓						
Prodigy		DCM, NIfTI/DCM, NIfTI (2D) + text data			✓	✓			✓
Ril-contour		NIfTI/NIfTI		✓ + auto segmentation			✓		

“Both” indicate cloud-based and non-cloud-based features. “*DCM*” is an abbreviation of DICOM files

It is noted to mention that some words and steps might have been used in different categories or instead of each other in AI data preparation studies. For example, some might categorize the “annotation” and “curation” as two separate categories in their studies [7], while the “annotation” is considered part of data curation in other studies [8]. Likewise, in the current work, we consider and describe these steps as subsets of data curation.

## Data Curation

**Fig. 1:**
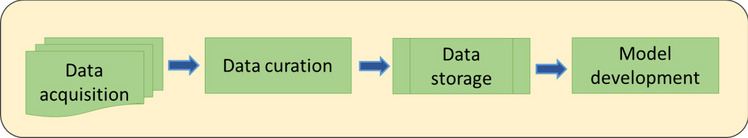
Workflow of required steps for proper model development

Data curation is an important process in model development, applied to data from the time it is first acquired to the point it is ready for use by AI (Fig. 1). Tools have been widely developed to address some or all of the steps for data curation [7].

**Fig. 2:**
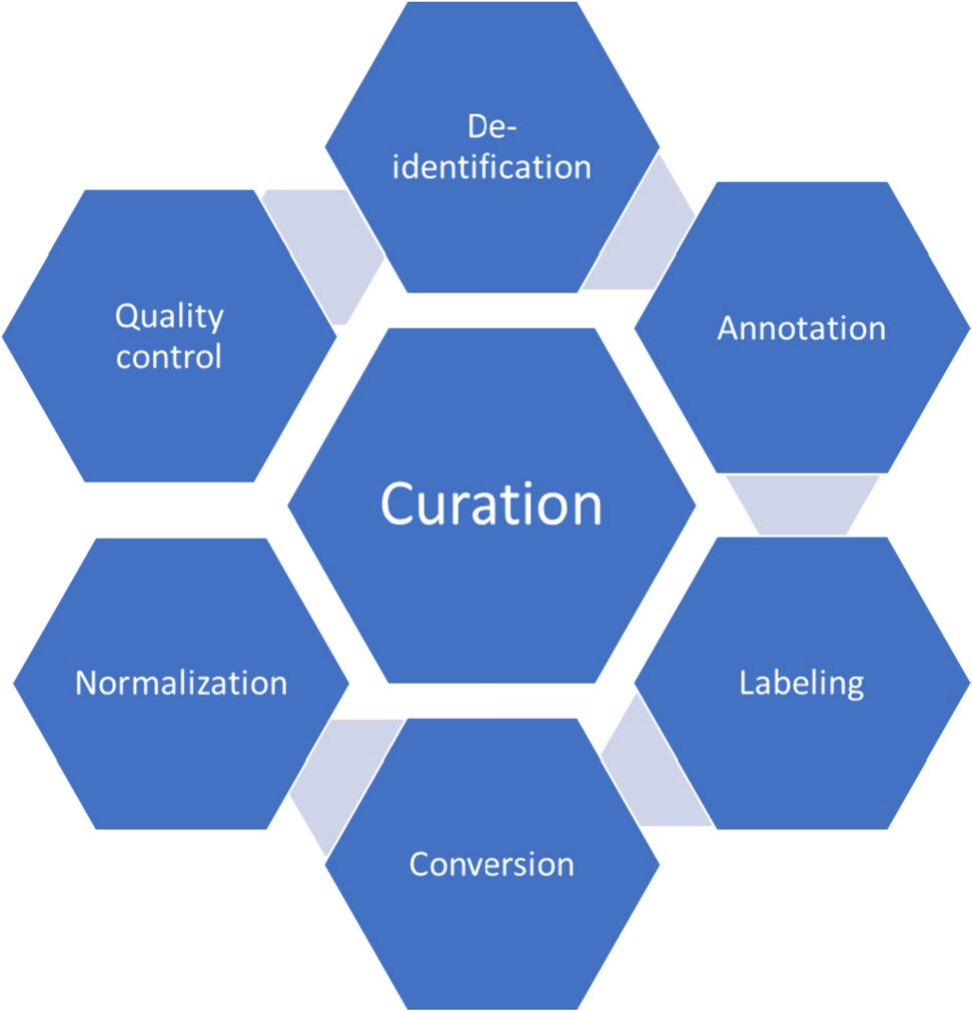
Demonstration of steps required for optimal data curation

Data curation can be referred to as the process of collecting, sorting, filtering, tagging, normalizing, standardizing, converting, and management of data prior to feeding the data to AI models for development purposes (Fig. 2). This broad category of tasks plays a crucial role in optimizing model development in the field of medical imaging [4].

## De-Identification

**Fig. 3:**
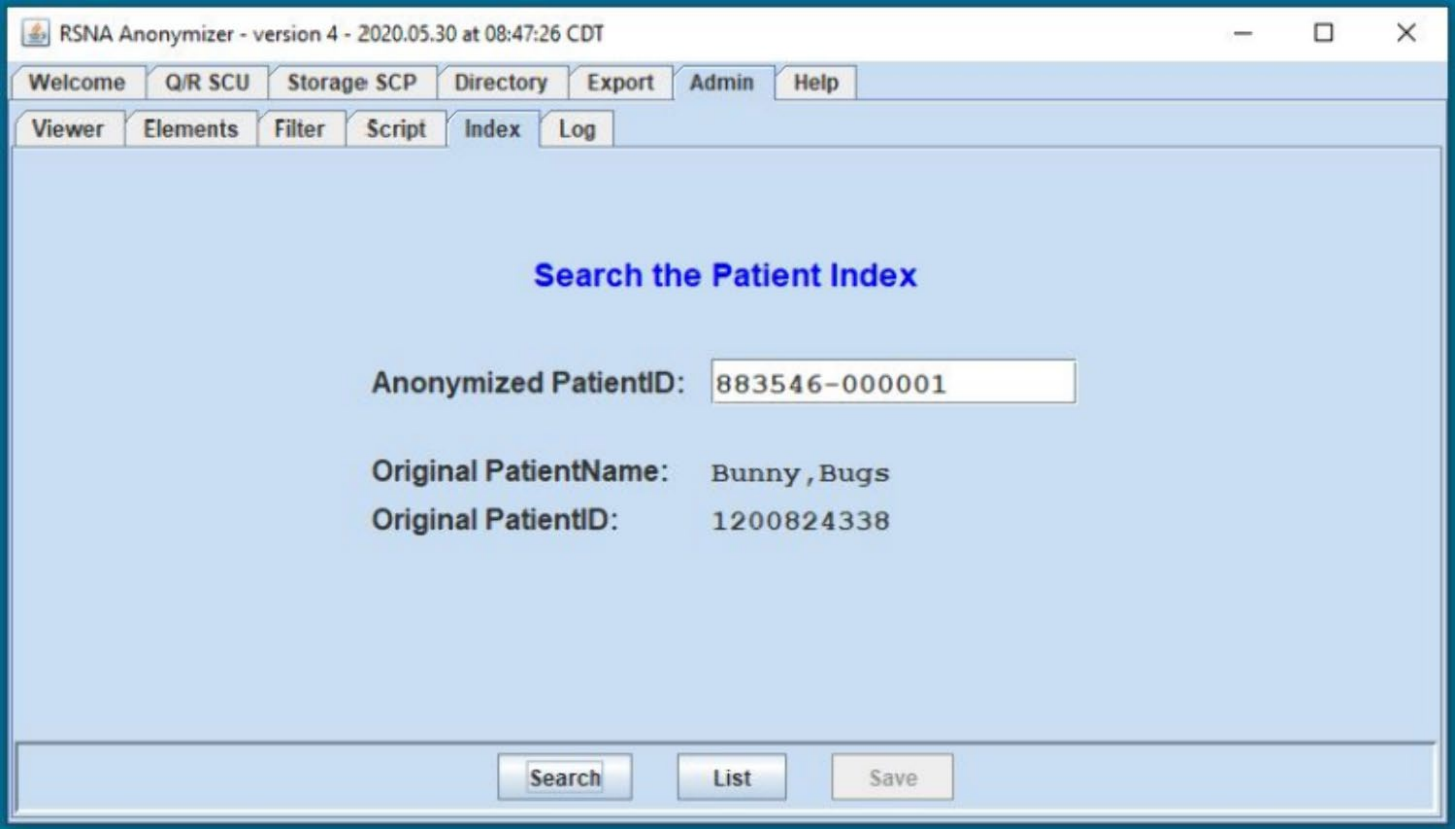
De-identification by RSNA anonymizer tool—the images uploaded from the path provided by the “Directory” tab or a PACS connected by the “Q/R SCU” tab are processed and exported to the output path by use of the “Export” tab; the images can be viewed in the “Viewer” tab, and their original identifiers can be tracked by the “Index” tab

In the United States, the Health Insurance Portability and Accountability Act (HIPAA) de-identification approaches, including “Safe Harbor” and “Expert Determination” delineates a comprehensive set of distinct categories of protected health information (PHI) that necessitate removal prior to the use of a medical document for many research endeavors [9]; most countries outside the US also have similar requirements for privacy preservation. It is worth noting that institutions have a range of de-identification methods to select from since there is not a concrete consensus on using a specific method. The data objects must undergo modifications that involve eliminating unnecessary PHI and substituting essential PHI with research identifiers. The research identifiers maintain the connection between data objects in addition to establishing a disassociation between the data and the human entity which served as the subject of the trial (Fig. 3). There are two types of de-identification [10]. One is “anonymization,” which replaces all PHI with either nothing or random data; anonymized data is completely devoid of any information that could potentially disclose the patient’s identity. The second approach is “pseudonymization.” In this approach, a known identifier replaces PHI, and there is a separate file that stores the mapping from this identifier to the PHI [11]. The latter approach enables researchers to conduct follow-up studies and additional multi-modal analyses in their future works and is common in clinical trials.

PHI is nearly always present in the meta-tags of medical images (e.g., DICOM header). Patient data can also be part of the image (pixels), which is called “burned-in” data. This may be introduced by post-processing software that puts PHI into the pixels, especially in cases of some older imaging devices. Specific tools are introduced to adhere to the mandates of HIPAA in addition to ensuring the preservation of data quality and reliability, but no gold standard tool for pixel-level de-identification has been introduced yet. In addition to textual PHI de-identification, it is possible to identify an individual based on the images themselves, such as facial reconstruction in neuroradiology imaging [12]. For this purpose, defacing tools such as “Mridefacer” [13] will alter voxels in the facial region of an MRI scan while preserving the brain structure. A list of data de-identification tools that are commonly applied for de-identification in our survey is demonstrated in Table 1.

## Data Format and Conversion

One of the initial considerations for choosing a tool is the “data format” that the user is working with. Digital Imaging and Communications in Medicine (DICOM) is known as the standard file format and communication profile in radiology [14]. The NIfTI file format was later developed to store volumetric image data, such as 3D MRI scans, in a standardized manner. It covers a wide array of 2D, 3D, and 4D data formats, including both structural and functional MRI, diffusion tensor imaging (DTI), and positron emission tomography (PET) scans [15]. NIfTI is used in medical imaging because it directly supports 3D and 4D data, while JPEG is primarily a 2D format (there is a 3D JPEG standard, but we are not aware of any annotation tool that supports it). Most deep-learning models require input in the form of matrices or tensors. This typically involves converting the DICOM images into a pixel array representation or converting them to a standard image format like JPEG, PNG, or NIfTI format.

If a tool only accepts JPG format, one can not work on NIfTI images with it. “ImageJ” is a tool that receives and saves several types of data, including JPEG, PNG, FITS (Flexible Image Transport System), TIFF (Tag Image File Format), and DICOM. One of the software that was widely used by our survey participants was the dcm2niix. It is exclusively developed for the conversion of DICOM files to NIfTI format, providing multiple options to use the original DICOM’s metadata in the naming of the output NIfTI file. This can be considered an advantage since converting DICOM to alternative formats can lead to losing metadata information related to images, which is not desired by users in many AI applications [16]. Converting tools mentioned in our survey can be found in Table 1.

## Image Normalization

In medical imaging, it refers to the process of adjusting the intensity values or pixel sizes of medical images to a more consistent scale or range. Normalization plays an important role in medical image curation as different imaging devices or techniques can produce images with varying intensity ranges, scales, and statistical distributions [6]. In this regard, many software have introduced tools for data normalization, such as “MONAI Label” and “Mango.” In addition, one important part of normalization is harmonization, which makes studies from various institutions consistent and compatible. In our survey, the “Medical Image and Data Resource Center” (MIDRC) was the only tool that harmonizes imaging studies through Logical Observation Identifiers Names and Codes (LOINC) mapping. It yields common long names and thus enables searching and efficient cohort building.

## Cloud Computing and Operating Systems

Cloud computing refers to the utilization of off-premise computing services and infrastructure provided by a separate entity for storing, managing, and processing medical image data. It involves the storage of medical images and related information on remote servers hosted in data centers over the Internet [17]. On the other hand, a non-cloud tool is executed on local hardware. Users must install the software on these local servers and continuously manage their maintenance, updates, and security [18]. This maintenance overhead, and also the upfront cost, may be incentivizing institutions to use cloud computing more for their AI development pipelines. However, cloud computing has some challenges in terms of operational management, interface efficiency, financial costs, and security preservations [19].

When choosing a suitable tool, developers should consider the operating system required by the tool as well. While most tools introduced in our survey are agnostic, meaning they run on any common operating system, some tools only work on specific operating systems. In our survey, “Horos,” which is used for data de-identification and labeling, works exclusively on Mac; on the other hand, “Niffler” and “Moose” are solely available for Linux operating systems.

Table 1, derived from our survey, illustrates the tools that exhibit the characteristics outlined above.

## Annotation and Labeling

Image annotation and labeling are often used interchangeably, but they are two different tasks commonly used for the analysis of medical imaging. Annotations represent the regions that contain the developer’s object of interest; they might be in the form of a bounding box or a freeform delineation around the portion of the image thought to represent the object or pathology of interest. Labels provide a textual or categorical representation of the identified regions or structures. Labeling is the process of classifying the images or the annotations into certain categories [20]. Labeling is required for most classification tasks, and annotation may also be required. For example, a chest radiograph can be labeled as “pneumonia” or “normal” without a need for annotating the suspected region; it can also be used in companion with an annotation task for labeling multiple pathologies annotated in an image, such as “consolidation,” “pneumothorax,” or “mass.” Table 2 demonstrates the core features of collected tools for annotation and labeling.

### Segmentation

**Fig. 4:**
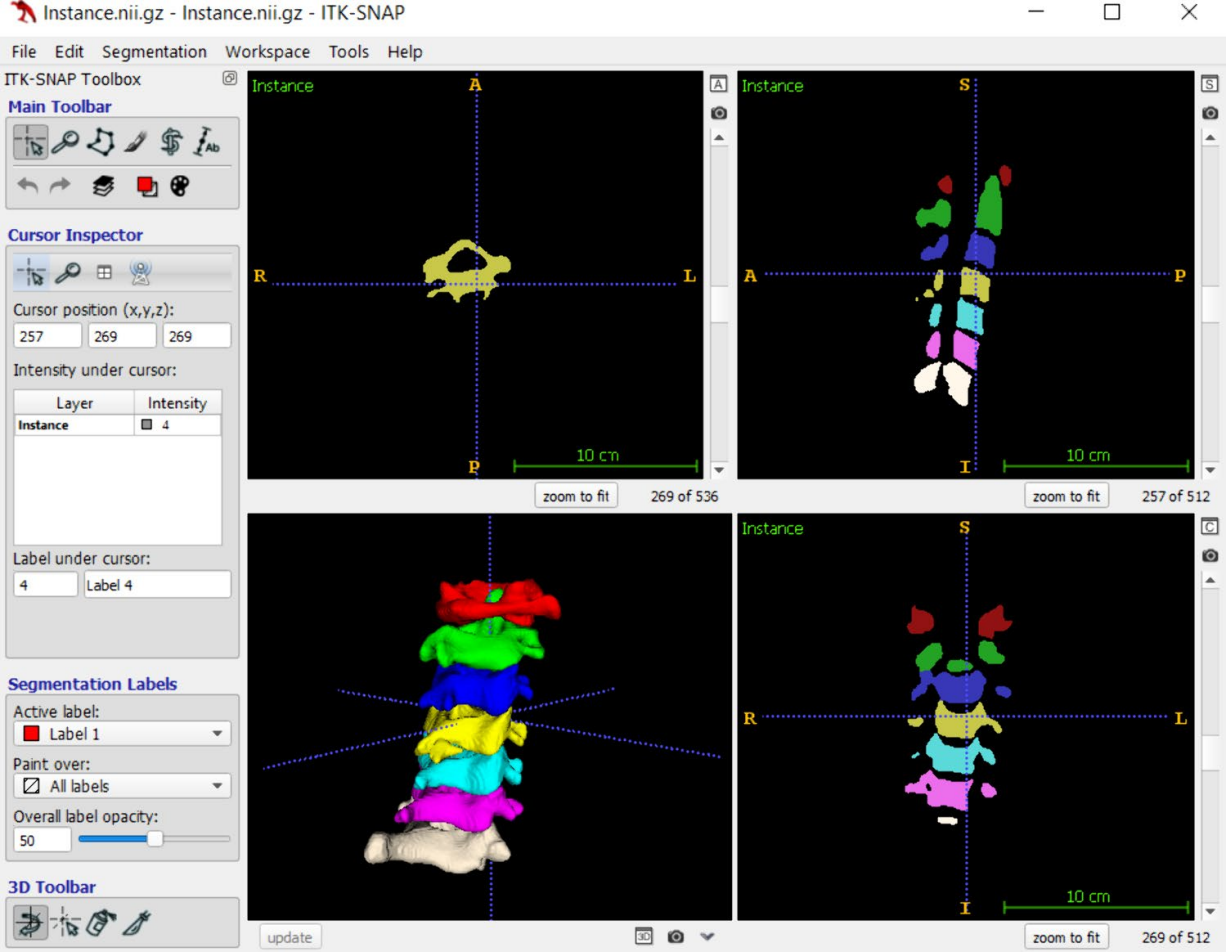
Multi-class segmentation and 3D rendering by ITK-SNAP software. Segmentation is performed by the “paint” tool on the provided medical image (hidden in the figure for visualization purposes). Users can use the color palette, edit with the “eraser,” shift between multiple labels through the “Active label” tool, and change the opacity of labels for better visualization. Multiple object masks can be saved and loaded as a single file, each object showing a unique anatomical region

Annotation tasks can be conducted by finely delineating the exact borders of an object, as in segmentation (Fig. 4). Segmenting a large number of data manually is tedious and time-consuming. Semi-automated and fully automated tools that facilitate the process of segmentation [21, 22] are available. “Semi-automated segmentation” is a combination of manual user input and automatic algorithms. In this approach, the user typically initiates the segmentation by providing an initial annotation or seed region in the image. The tool then applies algorithms to propagate and refine the segmentation based on the user-provided information, which can be adjusted and corrected by the user [23]. On the other hand, “fully automatic segmentation” tools do not require user input or intervention for explicit segmentation [24]. They analyze the image data and identify the regions of interest based on the learned template.

Since many of the image annotation tools are open-source, they benefit from constant improvement and expansion by new extensions being developed. A good example is the recently introduced tools of automatic segmentation, annotating several body organs with the click of a button. These tools, including MONAI bundle model zoo [25] and the “segment anything model (SAM)”-based medical imaging tool (MedSAM) [26], have introduced extensions to be used on popular tools used in our surveys such as 3D Slicer and OHIF; so that the operator can edit the generated annotations and extract different measures from the annotated masks.

### Object Detection

**Fig. 5:**
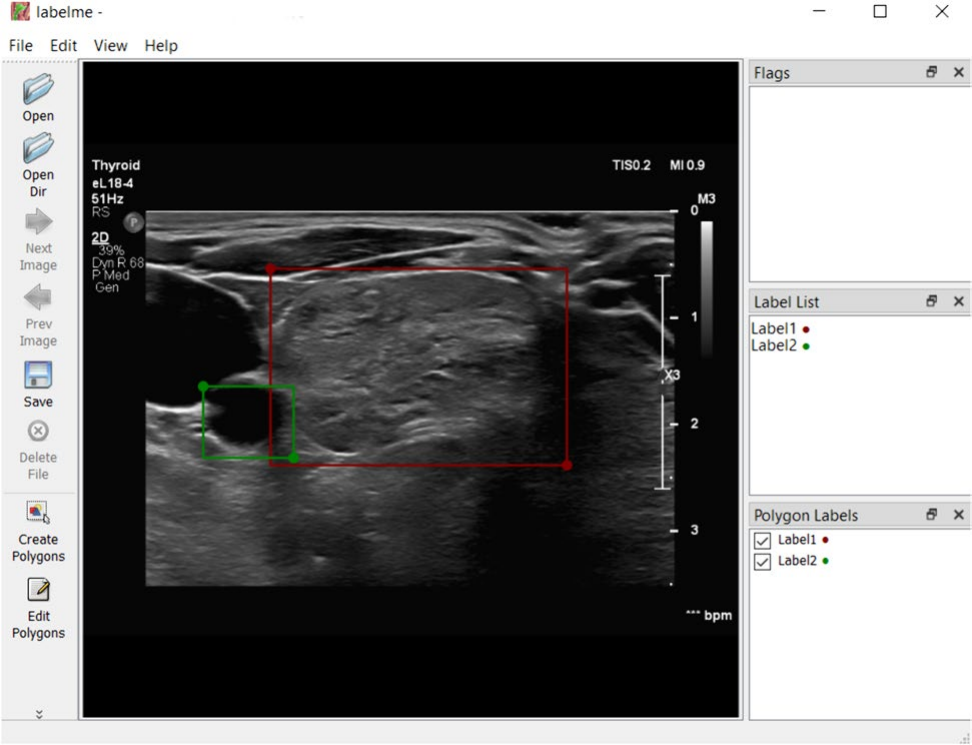
Bounding box generation by the “Labelme” software—two-point coordinates selected on an ultrasound frame (the green and red dot) are used to generate 2D rectangular boxes. The boxes’ center coordinates and dimensions in two axes are saved to be used as the “region of interest” or as labels for object detection tasks

It is a gross detection of an object without distinguishing its exact borders to enhance the training process by focusing on the region of interest and assigning a class label for that region [27]. This task can be performed by tools such as Label Me, Markit, or Prodigy. They store the coordinates of the manually generated, so-called bounding boxes in formats, such as JSON, to feed the deep learning object detection models (Fig. 5).

### Active Learning

It is another feature in segmentation that helps annotators by providing suggestions on challenging regions during annotation. They guide annotators to data points that would result in improvement of model performance. This can also be used to optimize segmentation performance over time after initial segmentation [28, 29].

### Co-registration

It is the alignment of two or more volumetric images based on specific mathematical transformations [30]; it provides multi-parametric information about the structure or region of interest [31, 32]. By aligning images, co-registration enables one to combine voxels for anatomical structures from different image types like functional MRI, T1-weighted, diffusion-weighted images (DWI), or pre- and post-contrast images [33]. 3D slicer, ITK-SNap, MITK, and ImageJ were applications that included co-registration functions in our survey.

Table 2, derived from our survey, demonstrates the tools that encompass the features outlined above.

## Data Collection and Storage

Medical imaging data are usually acquired by submitting a query from identified data sources such as Picture Archiving and Communications Systems (PACS). The query contains various search criteria such as patient demographics (e.g., age, gender), examination type (e.g., X-ray, MRI), body region (e.g., head, abdomen), imaging modality, and date range. Since most studies focus on a specific disease and since the PACS usually does not store diagnostic codes, it is often necessary to first query a diagnosis database or registry and then perform the PACS query.

“Atlas” is a platform to support design and observational analyses. It is used for phenotyping and cohort definitions for target, control, and outcome populations. Another platform for data retrieval is “DIANA,” which is capable of de-identification, cohort definition, and radiation dose monitoring for prospective oncology studies [34].

While nearly every radiology or imaging practice has a PACS, some open-source storage solutions for DICOM images were introduced in our survey. “Orthanc” is a standalone DICOM server that provides an extensible platform for storing, retrieving, and managing medical images. It can be easily integrated into existing PACS infrastructures. “MIDRC” exists on the Gen3 Platform for intake, storage, viewer, cohort building, and data downloading [35]. With the increasing size and complexity of medical imaging datasets, cloud computing platforms such as “NVIDIA DGX Cloud,” “AWS,” “Google Cloud,” or “Microsoft Azure” provide scalable infrastructure for data storage, processing, and training. These platforms offer a range of tools and services that facilitate data management, distributed computing, and access to powerful GPUs for accelerated AI training.

## Federated Learning

Obtaining ethical and legal approvals such as Institutional Review Boards (IRB) or data use agreements with healthcare research institutions is a prerequisite for starting data collection. With the rapid advances of AI in medical imaging, further collaboration in multi-institutional frameworks is a key component in eliminating biases and the development of reproducible and standardized models. This goal was previously only pursued by data sharing among institutions and involved significant difficulties, including limited data storage and data privacy challenges. Data privacy concerns or legal restrictions limit the accessibility and usability of the required data. Federated Learning was introduced as a possible solution in medicine [4]. Federated learning is a privacy-preserving approach in which the data is used locally to train a model, and the weights from the local training are sent to a central server. Then the updates from all sites are combined, and the new weights are sent back to all the sites. The weights from multiple institutions are iteratively updated and shared with the central model until its performance reaches the ending criteria [36]. Tools such as “NVIDIA FLARE” and “MLFlow” provide an environment for researchers to adapt existing AI model workflow to federated learning.

## Workflow

Data handling, model development, and performance evaluation are considered three requisites of building AI algorithms. Each of these tasks is divided into steps based on the data at hand and the general approach to model development. Establishing a workflow enables developers to gain an overview of the whole network, follow defined steps, and track their model’s performance in each task at hand. Platforms, such as MD.ai and MLflow, provide a workflow with a wide range of applications from data preparation to evaluation.

## Security Considerations

Radiology departments and AI developers must adhere to stringent data protection regulations ensuring the security and privacy of patient data. In addition to data de-identification mentioned above, there are other key considerations for securing patient data. “Data encryption,” “availability,” and “integrity” are major concepts for data security framework. Data must be encrypted both at rest and in transit. This ensures that even if data is intercepted or accessed without authorization, it remains unreadable and secure [37]. Backup strategies and data recovery solutions are essential components of a comprehensive security plan [38]. Cloud service providers should provide robust security measures, compliance with healthcare regulations, and features that support the secure handling of radiology images and associated patient data. Some examples of clouds following this structure are AWS, Microsoft Azure Health Data Services, and Google Cloud Healthcare API. In addition to the cloud services, there are open-access tools in the imaging informatics space, which can be integrated with non-cloud products to promote information security. “Wire Shark” [39], which points out to analyzing network traffic, “Shodan” [40], which discovers internet-connected devices, and “Nmap” [41], which maps the network and identifies possible unauthorized connections.

## Discussion

Continuous improvements in AI model developments may overcome obstacles of data curation for optimal data preparation. The challenging task of removing burned-in data or defacing during de-identification may eliminate parts of the metadata critical for advanced processing [42]. Khosravi et al. [43], in a recent work, developed a deep learning model to detect and anonymize radiographic markers. The AI tools for automatic segmentation have substantially improved. Many developers build emergent deep learning models to curate specific body parts or tumors. For example, Wasserthal et al. proposed a deep learning model entitled “Total Segmentator” for segmenting 117 anatomical structures [44]. In another study, Cai et al. introduced a deep learning model for the segmentation of intracranial structures in brain CT scans [45]. Furthermore, deep learning studies focusing on superresolution and mask interpolation based on voxel values are vastly under investigation. These techniques potentially improve the quality of medical images with low resolution and further reduce hallucinations in imaging reconstructions [46]. These developments may increase inter-reader agreements, creating a more feasible workspace for annotators in the future. No matter what the purpose, the optimal tool should have intuitive interfaces, clear workflows, and comprehensive documentation to facilitate its application. Furthermore, software support for these tools is desirable, like NVIDIA AI Enterprise [47], which supports MONAI, MONAI Label, and FLARE.

In addition to the aforementioned tools, platforms such as “MIDRC” provide researchers with data commons and valuable machine learning algorithms, including a metrology tree to help AI investigators determine appropriate performance metrics and a bias awareness tool to help AI investigators identify potential sources of bias and understand methods of mitigation [48]. Online competitions and challenges such as the Brain Tumor Segmentation (BraTS) Challenge [49] and the RSNA Kaggle Competition [50] have brought together developers and provided opportunities to showcase their expertise, collaborate with peers, and push the boundaries of medical image analysis and artificial intelligence. These platforms drive innovation–refining algorithms and advancing medical imaging for AI applications.

Training and support resources provided by tool developers or the research group community can also influence the process of choosing the appropriate tool. The particular preferences and skill levels of the users are important factors to be considered for optimizing their adoption and productivity.

In our study, we excluded commercial and not open-source tools despite covering a wide range of data curation tasks. For instance, MD.ai [51] was used by several participants of the survey as an image viewer, de-identifier, format converter, and segmentation tool, even though it is not open source. Additionally, imaging formats such as “Analyze” and “minc” [52], as well as co-registration tools such as Advanced Normalization Tool (ANT) [53], which are vastly known, were not mentioned by participants in the current study.

## Conclusion

This study provides a comprehensive overview of available open-source tools, drawing from the insights and experiences of the SIIM community. By curating a list of practical and widely used tools, we aimed to streamline the process of tool selection for researchers, enabling them to make informed decisions based on community expertise.

It is important to recognize the dynamic nature of the field of AI. As advancements continue to be made and new tools emerge, our work can serve as one of the starting points for researchers seeking to navigate the ever-expanding landscape of open-source tools. By incorporating feedback from the community and staying abreast of the latest developments in the field, we aim to continually improve the utility and relevance of our resources for researchers.

## Data Availability

The dataset collected is available upon reasonable request to the corresponding author.
